# Acquisition of an oncogenic fusion protein is sufficient to globally alter the landscape of miRNA expression to inhibit myogenic differentiation

**DOI:** 10.18632/oncotarget.19693

**Published:** 2017-07-29

**Authors:** Jacob M. Loupe, Patrick J. Miller, Judy S. Crabtree, Jovanny Zabaleta, Andrew D. Hollenbach

**Affiliations:** ^1^ Louisiana State University Health Sciences Center, Department of Genetics, New Orleans, LA 70112, USA; ^2^ Louisiana State University Health Sciences Center, Department of Pediatrics and Stanley S. Scott Cancer Center, New Orleans, LA 70112, USA; ^3^ Current/Present address: Center for Human Genetic Research, Massachusetts General Hospital, Richard B. Simches Research Center, Boston, MA 02114, USA; ^4^ Current/Present address: Tulane University, New Orleans, LA 70112, USA

**Keywords:** alveolar rhabdomyosarcoma, myogenesis, PAX3-FOXO1

## Abstract

The differentiation status of tumors is used as a prognostic indicator, with tumors comprised of less differentiated cells exhibiting higher levels of aggressiveness that correlate with a poor prognosis. Although oncogenes contribute to blocking differentiation, it is not clear how they globally alter miRNA expression during differentiation to achieve this result. The pediatric sarcoma Alveolar Rhabdomyosarcoma, which is primarily characterized by the expression of the PAX3-FOXO1 oncogenic fusion protein, consists of undifferentiated muscle cells. However, it is unclear what role PAX3-FOXO1 plays in promoting the undifferentiated state. We demonstrate that expression of PAX3-FOXO1 globally alters the expression of over 80 individual miRNA during early myogenic differentiation, resulting in three primary effects: 1) inhibition of the expression of 51 miRNA essential for promoting myogenesis, 2) promoting the aberrant expression of 43 miRNA not normally expressed during myogenesis, and 3) altering the expression pattern of 39 additional miRNA. Combined, these changes are predicted to have an overall negative effect on myogenic differentiation. This is one of the first studies describing how an oncogene globally alters miRNA expression to block differentiation and has clinical implications for the development of much needed multi-faceted tumor-specific therapeutic regimens.

## INTRODUCTION

Pathologists use the differentiation status of cancer cells as a prognostic indicator to predict the aggressiveness and stage of a given tumor. In general, a low level of differentiation, in which the tumor cells lose structural organization, grow more rapidly, and are morphologically dissimilar from the surrounding normal tissue, correlates to a higher level of aggressiveness and a poor prognosis. There are two basic mechanisms by which tumor cells can acquire an undifferentiated state. First, as shown with neuroblastoma and glioblastoma [1–3], the hypoxic state of a solid tumor can dedifferentiate cancerous cells into a less mature state. Second, the presence of an oncogene can actively block the differentiation process [4–6]. However, how the oncogene produces large-scale changes in gene and/or miRNA expression to achieve this state is not yet fully understood. Regardless of the mechanism by which tumor cells achieve the undifferentiated state, researchers have capitalized on this knowledge to develop differentiation therapy, in which undifferentiated tumor cells are induced to reactivate the differentiation program [7].

Like many other cancers, Rhabdomyosarcoma (RMS), one of the most common soft tissue sarcomas in children, is characterized by poorly differentiated muscle cells. RMS is comprised of two main subtypes: embryonal (ERMS) and alveolar (ARMS), each defined by its unique histology, clinical presentation, therapy, and prognosis [8]. ARMS, the more aggressive subtype, is primarily characterized by the t(2;13)(q35;q14) translocation, which creates the oncogenic fusion protein PAX3-FOXO1 [9, 10]. Among the mechanisms describing the impaired differentiation seen in ARMS [11–16], suppression of microRNAs (miRNA) miR-206 and miR-29 has been noted. MiR-206, one of the central miRNA that regulate myogenic differentiation, promotes myogenic differentiation, is downregulated in ARMS [17] and low miR-206 expression correlates to poor overall survival [18]. In contrast, silencing of miR-29 inhibits myogenesis [14]. Despite this knowledge, very little is known about how global miRNA expression levels change through the normal course of early myogenic differentiation and how the expression of PAX3-FOXO1 alters the landscape of changes during this same time period.

In this study we are one of the first to provide a picture of the global changes in miRNA expression that occur in the earliest stages of myogenic differentiation and to determine how the expression of PAX3-FOXO1 alters these changes, either directly or indirectly. We report that the oncogenic fusion protein mediates these changes in three distinct ways: 1) by inhibiting the expression of miRNA essential for promoting myogenesis, 2) by promoting the aberrant expression of miRNA not normally expressed during myogenesis, and 3) by altering the normal expression of miRNA. The ultimate result of these changes is predicted to be the inhibition of myogenic differentiation. Our results allow us to propose a model by which miRNAs contribute to the process of normal myogenic differentiation and to describe how the changes mediated by the oncogenic fusion protein would block normal myogenesis. Taken together, these results allow us to propose the use of miRNA-mediated differentiation therapy as a component of a multi-faceted treatment regimen for ARMS. Finally, the results presented here have implications that may be applied to other tumor models, since determining global changes in miRNA expression relative to the surrounding normal tissue may be used to identify key miRNAs that can be targeted for novel therapies for individual tumor models.

## RESULTS

### Effect of PAX3-FOXO1 on miRNA expression during early myogenesis

To determine how miRNA expression changes during early myogenic differentiation and to examine how PAX3-FOXO1 alters these changes, we utilized mouse primary myoblasts stably transduced with the MSCV-IRES-puromycin retroviral vector (negative control), or the same retroviral vector expressing FLAG-epitope tagged PAX3-FOXO1. The transduced cells were selected with puromycin; selected cells were harvested from three independent transductions and pooled, resulting in a single mixed population for each individual construct, which removes the potential for variability that may occur from clonal effects. The observed level of PAX3-FOXO1 expression is equivalent to that of the fusion protein in ARMS tumor cell lines [19, 20] and is therefore directly relevant to the role of the oncogenic fusion protein in ARMS.

**Figure 1:**
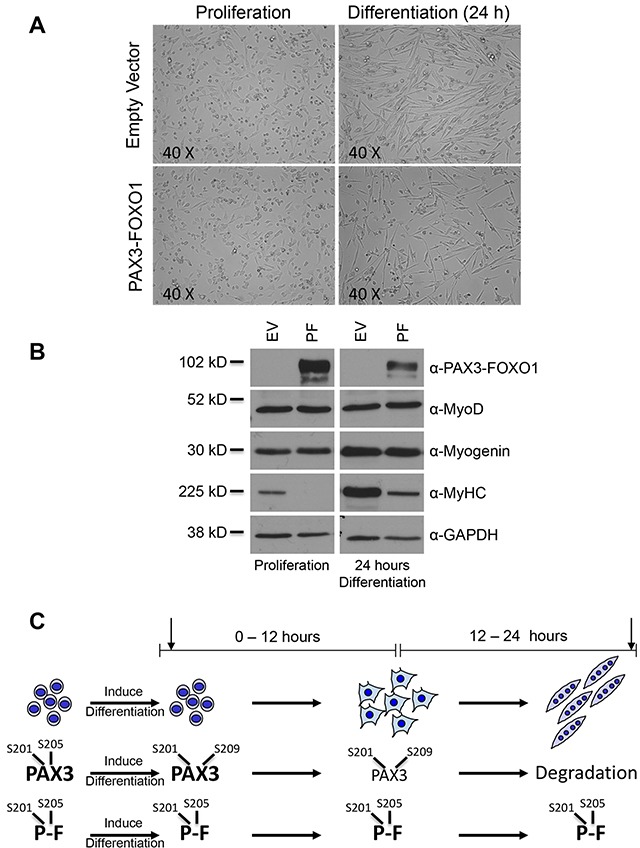
The stable expression of PAX3-FOXO1 inhibits terminal myogenic differentiation **(A)** Representative pictures of proliferating primary myoblasts (left panels) and myoblasts differentiated for 24 hours (right panels), either stably transduced with the empty vector negative control (top panels) or with the same vector containing PAX3-FOXO1 (bottom panels). **(B)** Total extracts were made from proliferating cells (left panel) or cells differentiated for 24 hours (right panel). In each panel the cells were stably transduced with empty vector (EV, left lanes) or the same vector containing PAX3-FOXO1 (PF, right lanes). Protein levels were determined by Western blot analysis using antibodies for PAX3-FOXO1, MyoD (early myogenic marker), Myogenin (mid-myogenic marker), Myosin Heavy Chain (MyHC, late myogenic marker), and GAPDH. **(C)** Schematic of normal myogenesis and the phosphorylation status of PAX3 and PAX3-FOXO1 in proliferating myoblasts and throughout the first 24 hours of differentiation. Phosphorylation sites are Serine 201 (S201), Serine 205 (S205), or Serine 209 (S209). The arrows indicate the time points at which miRNA expression was determined (30 minutes and 24 hours).

In this model proliferating cells were induced to differentiate, as described in the Materials and Methods, and allowed to differentiate for 24 hours. We found that the negative control cells effectively entered the myogenic program exhibiting flattened and elongated cells that have begun to organize, align and fuse into multinucleated myotubes (Figure 1A, top two panels). Proliferating cells stably expressing PAX3-FOXO1 were visually indistinguishable from the negative control cells. However, after 24 hours of differentiation, despite having a flattened and elongated appearance, these cells appeared disorganized and were unable to fuse into multinucleated myotubes (Figure 1A, bottom two panels). A Western blot analysis of markers of early (MyoD), mid (myogenin) and late (MyHC) myogenesis demonstrated that the expression of PAX3-FOXO1 had minimal effects on the expression of MyoD and myogenin but qualitatively decreased the expression of MyHC in both the proliferative state and after 24 hours of differentiation (Figure 1B). Taken together, these results demonstrate the effective differentiation of control cells in this model and that the expression of PAX3-FOXO1 allows the initiation of differentiation but inhibits the ability of the cells to fuse and achieve terminal differentiation.

Using this model, we performed miRNA deep sequencing analyses on total RNA isolated from three independent growths of stably transduced primary myoblasts. We selected time points based on the known phosphorylation status and protein expression levels of PAX3 and PAX3-FOXO1 during differentiation (Figure 1C). In the proliferative state, PAX3 is phosphorylated at Serines 201 and 205. Upon the induction of differentiation PAX3 undergoes a rapid (<15 minutes) loss of phosphorylation at Serine 205 with a gain in phosphorylation at Serine 209, changes in phosphorylation that do not occur on PAX3-FOXO1 [21–23]. Further, PAX3 protein levels begin to decrease after approximately 12 hours of differentiation and by 24 hours of differentiation are gone. In contrast, PAX3-FOXO1 is stable and does not undergo a similar loss in protein expression at 24 hours [24]. Therefore, we used cells from the proliferative state, the initial stages of differentiation (30 minutes; a time when changes in phosphorylation status are observed) and a point at which differentiation has progressed but terminal myotube formation has yet to occur (24 hours; a time when PAX3 expression is gone but PAX3-FOXO1 is stable). The resulting sequencing data was analyzed in large-scale comparative miRNA analyses using the miRNAKey program (see Materials and Methods). The data used for subsequent analysis were limited to miRNA displaying statistically significant differences (p < 0.05), as determined by the miRNAKey program, and miRNA present in all time points being analyzed. Further, we used changes in miRNA expression of ≥1.3-fold, either up- or downregulated, as the arbitrary cutoff for changes in miRNA expression.

**Figure 2:**
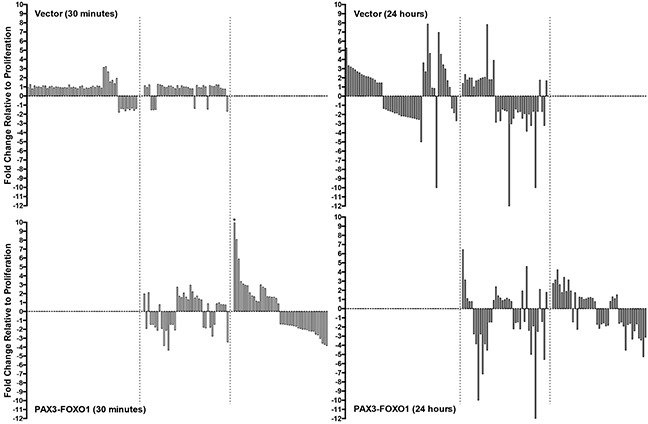
Waterfall plots illustrating the expression levels of miRNA in the negative control cells (Vector, top panels) and in cells stably expressing PAX3-FOXO1 (PAX3-FOXO1, bottom panels) at 30 minutes (left panels) and 24 hours (right panels) of differentiation The bars from left to right in each graph correspond to the miRNAs listed in order in Tables 1 – 3, respectively. All graphs are plotted on the same scale to facilitate the visual comparison of the magnitude of expression differences in individual miRNA. The asterisk indicates the one miRNA whose expression level (20.88 fold) falls outside the scale of the axis. The dotted lines delineate the three classifications of miRNA changes, as described in the Results.

We found that in the negative control cells 90 miRNA had changes in expression over the time course of differentiation while cells expressing PAX3-FOXO1 had 82 miRNA whose expression changed. Of these miRNA, 39 of the miRNA were found to have expression changes in both the control cells and in cells expressing PAX3-FOXO1 (Figure 2). Therefore, we are able to classify the miRNA into three categories: 1) miRNA whose expression is changed only in the vector only control cells (51 miRNA); 2) miRNA whose expression is changed only in the presence of PAX3-FOXO1 (43 miRNA); and 3) miRNA whose expression changes in both cell types (39 miRNA). Further, although 30 minutes may seem like a short period of time for changes in miRNA expression to occur, we found that 21/90 miRNA (23%) in the vector only control cells exhibited ≥1.3-fold increase or decrease in expression relative to the proliferative state at this time point (Figure 2). Interestingly the presence of PAX3-FOXO1 promoted more robust changes in miRNA expression at 30 minutes with 72/82 miRNA (89%) having ≥1.3-fold increases or decreases in expression, with nearly half of these miRNA changing over 2.5-fold (Figure 2). The results described here are part of a larger genomics study and as such the observed changes in miRNA expression were previously validated by qRT-PCR and published by us [19, 20].

A closer examination of the 51 miRNA whose expression changed only in the empty vector control cells revealed that they could be further categorized based on how their expression changed over the course of early differentiation (Table 1): 1) minimal change at 30 minutes relative to proliferation with increased expression at 24 hours; 2) minimal change at 30 minutes relative to proliferation with decreased expression at 24 hours; 3) increased expression at 30 minutes relative to proliferation with additional changes at 24 hours; and 4) decreased expression at 30 minutes relative to proliferation with additional changes at 24 hours. We identified validated gene targets for each of the miRNA using miRTarBase [25], using genes validated by two independent experimental methods. We performed a PubMed search on each of the validated target genes, using the gene name and the search terms “muscle” or “myogenesis” to determine if they were experimentally proven to be expressed in muscle and/or contribute to muscle differentiation. The miRNA and muscle target genes were analyzed through the use of QIAGEN's Ingenuity® Pathway Analysis (IPA®, QIAGEN, Redwood City, www.qiagen.com/ingenuity) to identify biological networks through which the targeted genes may functionally interact with one another. Finally, we performed a PubMed search to provide literature confirmation for the direct action of miRNA on target genes and/or the interaction of target genes with each other.

**Table 1:** miRNA with expression changes after 30 minutes and 24 hours of differentiation: changes seen only in cells stably transduced with the empty vector

miR	Target	Gene function	30 minutes	24 hours
**Minimal change at 30 minutes^#^ – increased expression at 24 hours**				
210-5p	E2F3COX1FBXO32	Proliferative transcription factorProstaglandin synthase – inhibits myogenesisExpression promotes muscle atrophy	−1.06	5.26
136-3p			1.26	3.34
33-5p	ABCA1	Influences intracellular cholesterol transport	−1.26	3.18
34c-3p	NCL	Maturation of ribosomes – inhibits Rb	1.14	3.04
133a-3p	NFATc4 Cdc42 Rhoa CCND2 SRF Pola1 Prdm16 Prkacb	Synergizes with MyoD to express myogenin Important for mitotic progression Downregulated in late myogenesis Proliferative control in G1 Essential for myoblast proliferation and fusion Initiation of DNA replication Promotes mitochondrial biogenesis Promote expression of early myogenic markers	−1.02	2.87
149-5p	E2F1ABCA1	Proliferative transcription factorInfluences intracellular cholesterol transport	1.02	2.68
450a-5p	HNRNPK	RNA processing – suppresses myogenesis	−1.06	2.56
434-5p	RTL1	Promotes muscle hypertrophy	1.12	2.36
^*^322-3p			1.12	2.25
542-3p	COX2	Prostaglandin synthase – inhibits myogenesis	−1.21	2.14
341-3p			1.02	2.12
483-5p	NCLSOCS3	Maturation of ribosomes – inhibits RbPromotes myogenesis – inhibits Jak/STAT	1.07	2.02
666-5p	ZEB2	Represses expression of E-cadherin	−1.09	1.90
409-3p			1.00	1.75
380-3p			−1.04	1.75
23b-3p	FBXO32 HES1 SMAD3 NOTCH1 ABCA1	Expression promotes muscle atrophy Target of NOTCH – inhibits expression MyoD Inhibits expression of myostatin Myoblast proliferation – inhibits myogenesis Influences intracellular cholesterol transport	−1.07	1.46
6944-3p			−1.11	1.38
**Minimal change at 30 minutes^#^ – decreased expression at 24 hours**				
151-3p			−1.08	−1.36
181d-5p	RUNX1	Activated by MyoD – promotes myogenesis	−1.12	−1.42
Let-7b-5p	MTPN	Growth factor that promotes muscle growth	1.21	−1.56
21a-5p	Pdcd4 Spry2 PTEN Reck Tgfbi	Overexpression promotes myogenesis Negatively affects FGF to promote myogenesis Loss promotes myogenesis Increase promotes ECM interactions in muscle ECM protein on surface of muscle fibers	−1.09	−1.63
93-5p	STAT3	Promotes later differentiation	−1.02	−1.72
28a-3p			−1.12	−1.85
183-5p			−1.26	−1.85
Let-7a-5p	IL13 Trim71 Lin28a IL6	Secreted by myotubes to recruit cells Associates with AGO2 to bind miRNA Disrupts maturation of miRNA Expression needed for fusion and myogenesis	1.03	−2.04
Let-7d-5p	BDNF	Neurotropic factor that promotes myogenesis	1.11	−2.08
Let-7i-5p			−1.29	−2.17
106b-3p			−1.12	−2.22
Let-7g-5p			−1.17	−2.27
17-5p	ESR1 CRIM1 HIPK3 TUSC2	Estrogen receptor – required for fusion Transmembrane protein expressed in muscle Enhances AR to promote myogenesis Promotes G1 arrest	−1.05	−2.32
92b-3p			1.08	−2.38
20a-5p	STAT3 PTEN SHOX2 VEGFA ULK1	Promotes later differentiation Loss promotes myogenesis Required for normal myogenesis Growth factor that induces myogenesis Promotes autophagy, required for myogenesis	−1.06	−2.43
196b-5p			1.10	−2.5
Let-7c-5p	Myc Lin28a EZH2	Inhibits MyoD activity and myogenesis Disrupts maturation of miRNA Represses expression of MyoD	1.08	−2.56
149-3p			−1.16	−5.00
**Increased expression at 30 minutes – changed expression at 24 hours**				
2137			3.13	3.65
466i-3p	CDC14A	Regulates exit from mitosis	3.22	2.66
667-3p			2.65	7.87
^*^503-5p	FGFR1 FGF2 WEE1 CDC14A AGO2 ATF6 CCNF Bcl2 CDC25A CHEK1 CCNE1 CCND1 CDKN1A CCNE2	Growth factor receptor, inhibits myogenesis Growth factor – inhibits myogenesis Regulates entry into mitosis Regulates exit from mitosis Part of RISC complex, miRNA maturation ER stress response – promotes apoptosis Cyclin F – G2/M arrest Anti-apoptotic protein Required for G1/S progression Phosphorylates CDC25A to delay cell cycle Cyclin E1 – regulates G1/S Cyclin D1 – regulates G1/S Inhibits G1/S progression Cyclin E2 – regulates G1/S	1.55	4.68
6356			1.73	−1.12
Let-7d-3p	CCND1	Cyclin D1 – regulates G1/S	1.36	−1.16
25-5p	CDKN1A	Inhibits G1/S progression	1.94	−10.00
**Decreased expression at 30 minutes – changed expression at 24 hours**				
139-5p	Kit Shc Jun IGF1R FOXO1 RUNX1	Receptor – promotes myogenesis from skin Adaptor protein for multiple growth factors Represses myogenic activity Promotes myogenesis Regulates proliferation – inhibits myogenesis Activated by MyoD – promotes myogenesis	−1.78	6.97
210-3p	NFkB1 Tcf7L2	Subunit of NFkB – inhibits myogenesis Promotes early, inhibits late differentiation	−1.36	4.58
^*^133a-5p	RhoA	GTPase detrimental to myogenesis	−1.40	3.43
351-3p			−1.63	2.98
501-3p	AR	Upregulates expression of MyoD/myogenin	−1.35	1.70
^*^181a-1-3p	AR DNMT3A	Upregulates expression of MyoD/myogenin Pro-myogenic DNA methyltransferase	−1.52	−1.02
10a-5p	BCL2l11 KLF4	Proapoptotic protein Promyogenic transcription factor	−1.32	−1.32
^*^181c-3p			−1.58	−1.82
27a-5p	RUNX1 Prpm15 PPARA CREB1	Activated by MyoD – promotes myogenesis Induces mitochondrial biogenesis Induces mitochondrial biogenesis Induces mitochondrial biogenesis	−1.37	−2.70

Fold expression changes indicate the miRNA gene expression at 30 minutes or 24 hours relative to levels seen in proliferating cells. The asterisk indicates microRNAs known to be myomiRs. ^#^Minimal change is considered to be miRNA expression between −1.30 and +1.30 fold change relative to proliferation levels.

**Figure 3:**
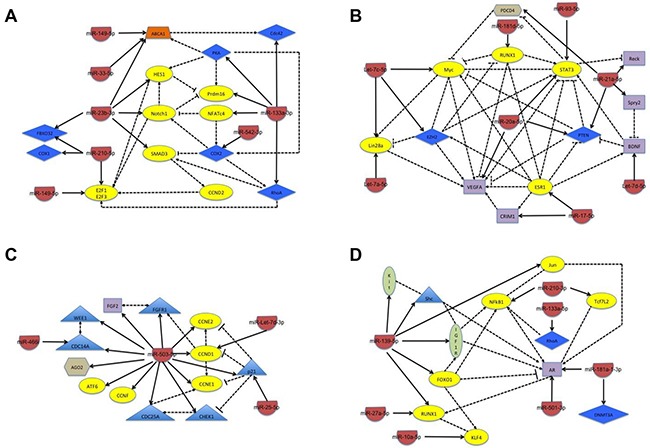
Schematic of the network of biological interactions between miRNA and their target genes whose change in expression is seen in the negative control cells only Schematics are separated into groups according to how changes occur during the first 24 hours of differentiation. **(A)** Minimal changes observed at 30 minutes with subsequent increase in expression at 24 hours of differentiation; **(B)** minimal changes observed at 30 minutes with subsequent decrease in expression at 24 hours of differentiation; **(C)** increased expression at 30 minutes with subsequent increase or decrease at 24 hours of differentiation; and **(D)** decreased expression at 30 minutes with subsequent increase or decrease at 24 hours of differentiation. The target genes are classified by color and shape according to their known biological function: yellow oval – transcription factor; blue diamond – enzyme; purple rectangle – ligand; green oval – receptor; orange tetrahedron – transporter; blue triangle – proliferative regulator; and brown hexagon – miscellaneous function. The solid line indicates the direct regulation of the expression of a target gene by the miRNA and the dotted line indicates a physical interaction between target genes, with both types of interactions being supported through literature evidence.

Consistent with the inverse relationship between miRNA and gene expression, we found that miRNA with minimal changes in expression at 30 minutes but *increased* expression at 24 hours of differentiation primarily target genes whose expression must *decrease* in order to allow myogenesis to progress (Table 1). These include genes whose expression inhibits myogenesis (e.g., RTL1, SMAD3, COX1 and 2, and Zeb2) and genes that regulate the expression of early myogenic markers (e.g., HES1 and NFATc4). Further, targeted genes also include those important for promoting cellular proliferation (e.g., E2F1, Notch1, and Cyclin D2), which must decrease to establish the post-mitotic state, and genes that regulate other biochemical functions required for myogenesis (ABCA1 and Prdm16). Network analysis highlighted biological interplay between many of the targeted genes (Figure 3A), which adds further downstream complexity to how miRNA inhibit the expression or activity of genes in order to promote myogenesis.

In contrast, the miRNA that exhibited minimal changes at 30 minutes but *decreased* expression at 24 hours of differentiation primarily target genes whose expression must *increase* for effective differentiation to occur (Table 1). These include genes whose expression promotes myogenesis and muscle regeneration (e.g., ESR1, RUNX1, STAT3, and VEGFA) or that inhibit the expression or activity of early myogenic markers (Myc and EZH2). Further consistent with the need of the post-mitotic state, TUSC2, which promotes G1 arrest, along with several other genes whose biological functions are necessary for the efficient progression of myogenic differentiation are targets (Table 1). Network analysis demonstrated that many of the target genes have biological effects and/or interactions on each other within this subcategory, highlighting an even more complex interplay to promote myogenesis (Figure 3B).

Interestingly, the miRNA whose expression increases at 30 minutes of differentiation primarily target cell cycle regulatory genes, many of which have pro-proliferative effects (Table 1 and Figure 3C). In particular, miR-503-5p, targets at least 14 known genes, most of which are essential for promoting cell cycle progression, including Cyclin D1, Cyclin E1, and CDC25A. Further, the miRNA in this subcategory have subsequent changes in expression at 24 hours that would further inhibit proliferation, including an additional increase in the expression of miR-503-5p, and a significant decrease in the expression of miR-25-5p, which targets the cell cycle inhibitory protein p21. Finally, as in other subcategories, there is additional biological interplay between target genes that would be expected to further promote the establishment of the post-mitotic state (Figure 3C).

Finally, miRNA with an initial decrease in expression at 30 minutes have changes that are consistent with the role the target genes play in early myogenesis (Table 1). For example, KLF4, IGF1R, and DNMT3A are all required for the initiation of myogenesis. However, their expression must decrease in the later stages of muscle differentiation, since they have been shown to inhibit myogenesis. Consistent with this fact, the miRNA that target these genes (miR-10a-5p, miR-139-5p, and miR181a-3p, respectively) have initial decreases in expression at 30 minutes with a concomitant increase in expression at 24 hours. Further, several miRNA in this subcategory maintain a decreased level of expression throughout the first 24 hours of differentiation, in particular miR-27a-5p, which targets genes important for mitochondrial biogenesis, a biological process essential for myogenic differentiation. IPA analysis further illustrates how biological interactions are predicted to occur such that increases in target genes at 30 minutes (e.g., IGF1R) would positively effect the expression or activity of additional factors essential for perpetuating myogenesis (e.g., AR) (Figure 3D).

**Figure 4:**
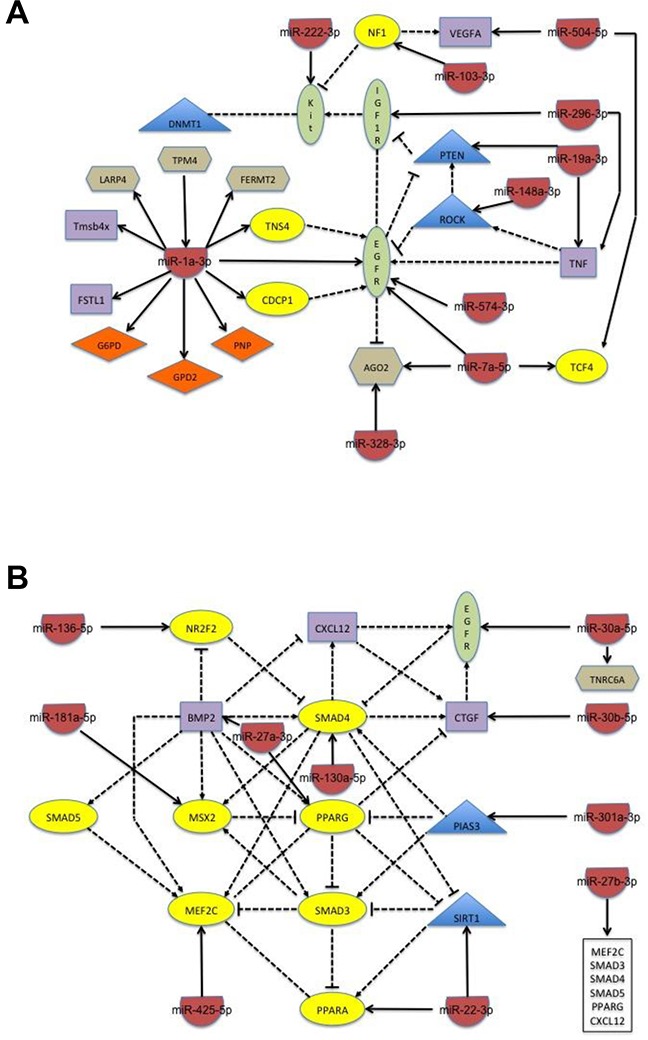
Schematic of the network of biological interactions between miRNA and their target genes whose change in expression is seen only in cells stably expressing PAX3-FOXO1 Schematics are separated into groups according to how changes occur during the first 24 hours of differentiation. **(A)** Increased expression at 30 minutes with subsequent increase or decrease at 24 hours of differentiation; and **(B)** decreased expression at 30 minutes with subsequent increase or decrease at 24 hours of differentiation. The target genes are classified by color and shape according to their known biological function: yellow oval – transcription factor; blue diamond – enzyme; purple rectangle – ligand; green oval – receptor; orange tetrahedron – transporter; blue triangle – proliferative regulator; and brown hexagon – miscellaneous function. The solid line indicates the direct regulation of the expression of a target gene by the miRNA and the dotted line indicates a physical interaction between target genes, with both types of interactions being supported through literature evidence.

A similar analysis of the miRNA whose expression changes occur only in the presence of PAX3-FOXO1 revealed that only two subclasses of changes were observed: 1) miRNA with increased expression at 30 minutes relative to proliferation with additional changes at 24 hours; and 2) miRNA with decreased expression at 30 minutes relative to proliferation with additional changes at 24 hours (Table 2). We found that the miRNA in the first subcategory target genes with apparently conflicting roles in myogenic differentiation, with miR-1a-5p effecting 11 of these target genes (Table 2 and Figure 4A). Consistent with the *increase* in miRNA expression at 30 minutes, several of the miRNA within this category target genes whose expression must decrease to allow the initiation of differentiation (e.g., miR-1a-5p targeting EGFR and miR-19a-3p targeting PTEN and TNF). However, in contrast, the maintained increase in several of the miRNA in this category target genes required to promote the fusion or terminal differentiation of muscle cells (e.g. TCF4, VEGFA, and TNS4). Taken together, and consistent with the pathological phenotype of ARMS tumor cells, alterations in the regulating miRNA would result in changes in target gene expression that would allow the initiation of myogenesis with a subsequent inhibition of myoblast fusion and terminal differentiation.

**Table 2:** miRNA with expression changes after 30 minutes and 24 hours of differentiation: changes seen only in cells stably expressing PAX3-FOXO1

miR	Target	Gene function	30 minutes	24 hours
**Increased expression at 30 minutes – changed expression at 24 hours**				
^*^1a-3p	CDCP1 TNS4 EGFR FERMT2 TPM4 LARP4 Tmsb4x FSTL1 G6PD GPD2 PNP	Receptor expressed a high levels in muscle Tensin – promotes expression of MyHC TKR – down regulation promotes myogenesis Scaffold protein– required for myoblast fusion Tropomyosin – involved in muscle contraction Cell morphology and cytoskeletal organization Chemoattractant – promote muscle regenerate Modulates actions of growth factors Involved in oxidative pentose pathway Involved in glycerol-phosphate shuttle Purine nucleoside phosphorylase	20.88	2.76
7a-5p	AGO2 TCF4 EGFR	Involved in miRNA maturation Transcription factor –MyHC expression TKR – down regulation promotes myogenesis	8.08	3.15
148b-3p	DNMT1	DNA methyltransferase; promotes myogenesis	5.89	4.26
328-3p	AGO2	Involved in miRNA maturation	3.38	2.63
148a-3p	ROCK1	Rho kinase that inhibits myogenesis	3.08	1.77
19a-3p	TNFPTEN	Inhibits myogenesis and MyoD expressionLoss promotes myogenic differentiation	2.95	3.43
532-5p			2.90	1.89
574-3p	EGFR	TKR – down regulation promotes myogenesis	2.11	3.15
296-3p	TNFIGF1R	Inhibits myogenesis and MyoD expressionTK receptor that promotes myogenesis	1.75	1.94
423-3p			1.68	−1.45
103-3p	GPD1 NF1	Carbohydrate and lipid metabolism RasGAP – loss inhibits myotube formation	1.17	1.75
^*^125b-5p	PPP1CA TRAF6	Phosphatase – energy production in muscle Activity required for myogenesis	1.09	−2.27
**Increased expression at 30 minutes – minimal change at 24 hours^#^**				
676-3p			3.00	1.27
674-3p			2.74	1.24
504-5p	TCF4 VEGFA	Transcription factor –MyHC expression Growth factor induces myogenesis	2.60	1.02
34b-5p			1.68	1.08
484			1.66	1.19
130b-5p			1.61	1.24
1983			1.61	1.14
^*^222-3p	KIT	TKR that promotes myogenesis from stem cells	1.48	0.77
**Decreased expression at 30 minutes – changed expression at 24 hours**				
27a-3p	RUNX1 PPARG FBXW7 BMP2 BMPR1A	Promotes myofibrillar disorganization Expression required for myogenesis Ubiquitin ligase targets Notch and Jun Inhibits myogenesis to induce osteogenesis Receptor – promotes satellite cell proliferation	0.86	−1.72
130a-3p	MEOX2 SMAD4 AGO2	Transcription factor, activates Myf5 expression Promotes myogenesis and fusion Involved in miRNA maturation	−1.41	−2.17
425-5p	MEF2C	Myogenic transcription factor	−1.42	−1.67
30a-5p	TNRC6A EGFR BDNF Snai1 CCNE2 MDM2 TIMP3	Downregulation required for myotube fusion Downregulation triggers myogenesis Promotes myogenic differentiation Required for initiation fo myogenesis Proliferative control in G1 Inhibits transcriptional activity of MyoD Blocks myotube formation	−1.45	−1.56
30b-5p	CTGF	Inhibits myogenesis by promoting fibrosis	−1.49	−1.88
872-3p			−1.54	−1.82
301a-3p	PIAS3 NKRF	Inhibits JAK/STAT – promote myogenesis Repression NFkB – inhibits myogenesis	−1.56	0.81
^*^181b-5p	TIMP3	Inhibits MMP and blocks myoblast fusion	−1.61	1.30
872-5p			−1.67	1.09
191-5p	MXI1	Inhibits Myc activity to promote myogenesis	−1.85	1.52
148b-5p			−1.92	−1.61
708-3p			−2.00	−1.47
^*^181a-5p	MSX2	Inhibits myogenic differentiation	−2.00	−1.92
22-3p	Cdc25c SIRT1 PPARA	Phosphatase controls entry into mitosis Stimulates myogenesis by activating Wnt Promotes mitochondrial biogenesis	−2.04	−4.54
^*^181c-5p			−2.17	−1.75
^*^27b-3p	RUNX1 Mef2C SMAD3 SMAD4 SMAD5 PPARG Cxcl12	Prevents myofibrillar disorganization Myogenic marker – promotes myogenesis Inhibits expression of myostatin Important for terminal differentiation Inhibits myogenesis – promotes osteogenesis Expression required for myogenesis Chemokine that promotes myoblast fusion	−2.22	−1.61
^*^24-2-5p	PRKCA	Inhibits cell spreading	−2.22	−3.33
541-5p			−2.56	−2.43
1957a			−2.63	−1.67
^*^208b-3p			−3.03	−3.22
148a-5p			−3.57	−3.44
30d-3p			−3.70	−5.26
136-5p	NR2F2	Orphan receptor – activates Myf5 expression	−3.84	−3.13

Fold expression changes indicate the miRNA gene expression at 30 minutes or 24 hours relative to levels seen in proliferating cells. The asterisk indicates microRNAs known to be myomiRs. ^#^Minimal change is considered to be miRNA expression between −1.30 and +1.30 fold change relative to proliferation levels.

The miRNA in the second subcategory, in which miRNA expression decreases at 30 minutes and remains decreased at 24 hours, for the most part exhibited small changes with a majority of them having between 1.5 – 2.0 fold decrease in expression (Table 2). The observed decrease in miRNA would subsequently result in an increase in the expression of target genes that are required to promote early myogenesis (e.g., MEF2C, SIRT1, and MEOX2). Further, these observed changes in miRNA expression would also promote the expression of genes that either inhibit myoblast fusion (e.g., RUNX1 and TIMP3) or inhibit terminal differentiation through the promotion of osteogenesis or fibrosis (e.g., BMP2, MSX2, and CTGF). Despite the smaller changes in miRNA expression, the effected target genes exhibit a complex interplay of biological interactions that would be expected to further allow the initiation of myogenesis with an inhibition of terminal differentiation (Figure 4B).

Although 39 of the identified miRNA demonstrated changes in expression in both the control cells and cells expressing PAX3-FOXO1, only 6 of these miRNA exhibited similar changes in expression between the two samples (Table 3). The remaining 33 miRNA had different patterns of expression in cells stably transduced with PAX3-FOXO1 relative to the empty vector control and could be categorized as follows (Table 3): 1) Increased expression from 30 minutes to 24 hours in both cell types with greater increases in cells expressing PAX3-FOXO1; 2) Increased expression from 30 minutes to 24 hours in control cells with decreased expression in cells expressing PAX3-FOXO1; 3) Decreased expression from 30 minutes to 24 hours in both cell types with higher levels of expression in PAX3-FOXO1 cells relative to control; and 4) decreased expression from 30 minutes to 24 hours in both cell types with lower levels of expression in PAX3-FOXO1 cells relative to control.

**Table 3:** MicroRNA changes that occur in cells stably transduced with empty vector (Vector) or PAX3-FOXO1 (PF)

miRNA	Vector30 min	Vector24 hours	PF30 min	PF24 hours	Target	Function
**Vector increased 30 minutes to 24 hours – PF appreciably increased relative to vector control**						
335-5p	1.13	1.39	1.98	6.45	RB1	Regulation of growth; decrease required for differentiation
^*^322-5p	−1.06	2.38	−1.92	3.14		
**Vector increased 30 minutes to 24 hours – PF decreased 30 minutes to 24 hours**						
PF – 30 minutes increase relative to EV – 24 hours decrease						
128-3p	1.24	1.76	2.13	1.11	Ppara Runx1 Pax3	Promotes mitochondrial biogenesis Promotes myofibrillar disorganization Expression must decrease for differentiation
PF – 30 minutes similar to EV – no increase at 24 hours						
3107-5p	−1.52	2.01	−1.47	0.79		
^*^486-5p	−1.52	1.99	−1.47	0.79		
PF – 30 minutes similar to EV –decrease at 24 hours						
10b-5p	−1.49	1.02	−1.75	−2.77		
PF – 30 minutes lower than EV – 24 hours further decrease						
434-3p	1.28	1.68	−2.13	−3,85	Vcan Ctnnb1	Versican – clearance facilitates myoblast fusion Beta catenin – activates expression of MyoD
^*^499-5p	1.23	1.76	−1.28	−10.00	Sox6	Required for the initial steps of differentiation
411-5p	1.15	1.94	−1.92	−2.78		
381-3p	−1.03	2.02	−3.84	−7.14		
410-3p	−1.06	2.07	−2.13	−3.85		
PF – 30 lower than EV – 24 hours stays constant						
^*^206-3p	1.10	7.82	−4.35	−4.54	Pola1 Pax7 Notch3 Fzd7 Meox2 Rarb Adar	Initiation of DNA replication Early myogenic marker of satellite cells Decrease needed for differentiation to progress Promotes expansion of myogenic satellite cells Activates expression of myf5 and is upstream of Pax3 Assists in initiating myogenesis Suppresses apoptosis upon differentiation
127-3p	1.10	1.81	−1.47	−1.47		
298-5p	−1.04	1.81	−1.47	−1.47		
PF – increase between 30 minutes to 24 hours – both appreciably lower than EV						
^*^133b-3p	−1.23	3.92	−2.08	−1.09	Pitx3 FOXL2	Controls ROS on shift to differentiation Downregulation required for late myogenic progression
**Vector decreased 30 minutes to 24 hours – PF appreciably increased relative to vector**						
PF – 30 minutes increase relative to EV – 24 remains constant						
320-3p	1.15	−2.86	2.74	2.40	Hspb6 E2F1	Heat shock protein involved in muscle function Proliferative transcription factor
25-3p	−1.20	−1.67	1.71	1.41		
PF – 30 minutes increase relative to EV – 24 hours decrease to proliferation levels						
Let-7e-5p	1.13	−2.70	1.57	1.17	Trim71	Associates with AGO2 to bind miRNA
29a-3p	1.00	−1.43	2.08	−1.12	Dnmt3B Dnmt3A Fbn1 Col3A1 Col2a2 Col1a1 Col4a1 Col4a2 Col4a3 Col4a4 Col4a5 Col4a6 Dcx	Establishes methylation patters to promote myogenesis Establishes methylation patters to promote myogenesis Fibronectin – structural protein in muscle Collagen present in muscle Collagen present in muscle Collagen present in muscle Collagen present in muscle Collagen present in muscle Collagen present in muscle Collagen present in muscle Collagen present in muscle Collagen present in muscle Required for myofiber maturation
34a-5p	−1.01	−1.56	1.62	−1.01	Bcl2 Sirt1 Notch1 Dll1 Vcl Pofut1 Bcl6 Gas1 CCND1 TGIF2	Anti-apoptotic protein Stimulates myogenesis by activating Wnt Inhibits myogenic differentiation Ligand of Notch1 – insures sustained differentiation Vinculin – a muscle structural protein Fucosylates Notch to optimize signaling Inhibit differentiation dependent apoptosis Growth arrest protein – promotes myogenesis Proliferative control in G1 Inhibits TGFbeta (which inhibits differentiation)
Let-7f-5p	−1.07	−1.64	1.32	1.15		
672-5p	−1.23	−12.50	2.96	1.03		
^*^221-3p	−1.25	−3.03	2.23	−1.25	Psmd9 Bnip3 Kit	Assembles 26S proteasome – required for fusion Inhibits Bcl2 Promotes myogenesis from stem cells
3968	−1.37	−2.43	1.52	−1.35		
PF – 30 minutes increase relative to EV – 24 hours decrease to EV levels at 24 hours						
423-5p	1.17	−1.41	1.74	−1.54		
31-5p	−1.07	−1.75	1.41	−1.45	Hif1a	Represses myogenesis by inhibiting Wnt
92a-3p	−1.11	−1.69	1.30	−2.22	Trp63 Tbx3	Required for later stages of myogenesis Transcription factor that inhibits myogenesis
PF – decrease 30 minutes relative to EV – 24 hours increase						
196a-5p	1.16	−2.43	−1.78	1.94	Hmga2	Required for late myogenic progression
16-5p	1.01	−1.92	−1.85	−1.42	Wnt3a CCND1 Bcl2 Vegfa Jun Jag1 CCNE2 FGF2	Promotes myogenic differentiation Proliferative control in G1 Anti-apoptotic protein Induces and promotes myogenesis Expressed in myotubes Receptor for Notch1 – inhibits myogenesis Proliferative control in G1 Promotes proliferation/inhibits differentiation of myblasts
335-3p	−1.45	−3.84	−1/14	4.61	Ank3	Expressed after differentiation; required muscle function
**Vector decreased 30 minutes to 24 hours – PF appreciably decreased relative to vector control**						
155-5p	1.16	−1.96	−1.78	−2.38	Cebpb Rhoa Fadd Reb Socs1 Maf Pea15a Lpin1	Inhibits myogenic differentiation GTPase detrimental to myogenesis Noncanonically required for myogenesis Negatively affects myogenesis Promotes myogenic differentiation Protein whose expression is activated by MyoD Negative regulator of apoptosis Promotes myogenic differentiation
21a-3p	1.06	−3.22	−2.78	−50.00		
28a-5p	1.06	−1.67	−1.45	−1.89	Srsf1	RNA splicing factor
**Similar changes (increased or decreased) between vector and PF from 30 minutes to 24 hours**						
92a-1-5p	1.24	−10.00	−1.13	−12.50		
344d-3p	1.21	−1.67	−1.02	−2.50		
351-5p	−1.16	1.75	−1.28	2.12	TNF E2F3	Inhibits expression of mid to late myogenic markers Proliferative transcription factor
98-5p	−1.29	−1.69	−1.28	−1.42		
^*^125b-1-3p	−1.31	−3.22	−1.35	−5.56		
^*^133b-5p	−1.67	1.69	−3.44	1.80		

Fold expression changes indicate the miRNA gene expression at 30 minutes or 24 hours relative to levels seen in proliferating cells in cells stably transduced with the empty vector (Vector) or with vector containing PAX3-FOXO1 (PF). The miRNA are separated into individual patterns based on their changes in expression during this time period. The asterisk indicates microRNAs known to be myomiRs. ^#^Minimal change is considered to be miRNA expression between −1.30 and +1.30 fold change relative to proliferation levels.

Examination of the miRNA whose expression changes in both cell types demonstrate that the alterations seen in the presence of PAX3-FOXO1 would effect target gene expression in such a way as to inhibit myogenic differentiation (Table 3). For example, miR-206-3p targets genes that are necessary for maintaining cells in the early myogenic state (Pax7, Fzd7, and Meox2) while also targeting genes necessary for initiating myogenesis (Rarb) or genes whose expression must decrease for myogenesis to progress (Notch3). The changes in miR-206-3p in the negative control (a large *increase* at 24 hours of expression) would result in target gene expression favorable for promoting myogenesis. In contrast, the presence of PAX3-FOXO1 promotes a large *decrease* in this miRNA, subsequently increasing the expression of these target genes thereby inhibiting myogenic differentiation. Similar alterations are predicted to negatively effect many biological processes essential for proper myogenesis, including organization of myofibers (miR-128-3p/Runx1 and miR-29a-3p/Dcx), myoblast fusion (miR-434-3p/Vcan, miR-133b-3p/FOXL2, and miR-221-3p/Psmd9), expression of myogenic structural proteins (miR-29a-3p/multiple collagens, miR-34a-5p/Vcl, and miR-335-3p/Ank3), and the initiation of myogenesis (miR-499-5p/Sox6, miR-29a-3p/DNMT3B and 3A, miR-34a-5p/Sirt1, and miR-221-3p/Kit).

## DISCUSSION

The extent of differentiation exhibited by cells within a solid tumor is indicative of the behavior of that tumor, in which more undifferentiated cell types usually elicit a more aggressive tumor with a poorer patient prognosis [26]. In this report we demonstrate for the first time that the presence of an oncogenic fusion protein, PAX3-FOXO1, is sufficient to globally alter the expression of miRNA during early muscle differentiation relative to the normal control. The nature of these changes is such that their effects on target genes would be predicted to allow the initiation of cells into the myogenic program while inhibiting fusion and terminal differentiation into myotubes (Tables 1 – 3), which is consistent with our experimental model (Figure 1A and 1B). Since the only difference that exists relative to the normal control cells is the stable expression of PAX3-FOXO1, we conclude that the presence of the oncogenic fusion protein is one of the major contributors driving the undifferentiated tumor phenotype in ARMS.

The results presented here allow us to develop a model by which miRNA changes promote normal myogenesis and by which PAX3-FOXO1 promotes the undifferentiated state characterizing ARMS tumor cells. In normal primary myoblasts we observed 90 individual miRNA with altered expression, each having differing temporal changes in expression over the first 24 hours of differentiation (Tables 1 and 2). A correlation of these miRNA to their validated target genes suggested that these temporal changes would promote the proper progression through myogenesis. For example, miR-133-3p targets several genes (NFATc4, Cdc42, Rhoa, CCND2, SRF, and Prkacb [Table 1]) whose expression must decrease in order for myogenesis to progress. Consistent with this fact, miR-133-3p shows minimal changes in expression at 30 minutes with nearly 3-fold increase by 24 hours, thereby being predicted to decrease the expression of these target genes. Similarly, miR-29a-3p targets genes that either perpetuate myogenesis (Dnmt3B and 3A) or are structural genes necessary for the functioning of mature myofibers (Fbn1, Dcx, and Collagen family members) (Table 3). Therefore, the decrease in the expression of miR-29a-3p would be predicted to increase the expression of these genes important promoting later myogenesis. Taken as a whole, our work presents one of the first demonstrations of the temporal changes of miRNA expression during early myogenic differentiation.

Upon the random and somatic acquisition of the t(2;13)(q35;q14) translocation, an event mirrored in our *in vitro* model system through the stable transduction of primary myoblasts, the subsequent expression of PAX3-FOXO1 initiates a cascade of direct and indirect downstream events that globally alters miRNA expression, ultimately reprogramming differentiating myoblasts. These changes manifest in three ways: first, the expression of PAX3-FOXO1 *prevents* the normal changes in expression of 51 miRNA that target many genes essential for positively promoting myogenesis. Second, the fusion protein *promotes* changes in expression of 43 miRNA not normally changed during differentiation, targeting many genes that would either maintain cells in the early stages of myogenesis or would inhibit the myogenic process. Finally, the presence of PAX3-FOXO1 *alters* the expression of 39 miRNA relative to normal cells in such a way that these changes would be predicted to negatively affect the myogenic process. Taken together, the overall result of the global PAX3-FOXO1-induced changes in miRNA expression would be to inhibit terminal differentiation and maintain cells in the earlier stages of myogenesis, a prediction that is consistent with our experimental model (Figure 1).

In addition to providing a better understanding of the molecular aspects of how PAX3-FOXO1 contributes to the undifferentiated phenotype of ARMS tumor cells, our results also have clinical implications. A closer examination of our results provides an explanation why present targeted therapies are proving ineffective in clinical trials. Two of these drugs, Cixutumumab and Bevacizumab, each target a single gene that controls important biological processes for tumor progression: cellular proliferation by inhibiting IGF1R and vascularization by inhibiting VEGFA, respectively. Although initially promising, these therapies have not proven effective in Phase I or Phase II clinical trials for ARMS [27–30]. In light of the results presented here, this is not surprising given that in addition to being involved in proliferation and vascularization, IGF1R and VEGF are also important for initiating myogenesis and promoting terminal differentiation [31–34]. We found that PAX3-FOXO1 alters the expression of miRNA in such a way that would be predicted to decrease the expression of both IGF1R and VEGFA (Tables 1 – 3), thereby inhibiting differentiation. Therefore, although being effective in targeting tumor phenotypes in proliferating cells, these two drugs would further decrease the effectiveness of already lowered levels of IGF1R and VEGF, thereby exacerbating the undifferentiated phenotype and the aggressiveness of tumor cells.

Finally, the results presented here have implications for the development of novel therapies for the treatment of ARMS. We previously proposed the use of a multi-faceted regimen that targets genetic, molecular, or biological characteristics of ARMS. This proposed regimen included LiCl to target phosphorylation of PAX3-FOXO1, chloroquine to target the aneuploid state, and Cixutumumab to target growth factor related proliferation [20]. However, the present work argues against the inclusion of Cixutumumab. Instead, we propose to replace the use of Cixutumumab with targeting of select miRNA whose expression is altered by the presence of PAX3-FOXO1. The inhibition or replacement of microRNAs are growing in interest for use as potential cancer therapies [35], in particular as potential targets for differentiation therapy in solid tumors such as ARMS [7]. When combined with LiCl and chloroquine, this multi-faceted regimen would inhibit the activity of PAX3-FOXO1 [36], promote the death of aneuploid cells, which is induced by the presence of the oncogenic fusion protein [20], and potentially reactivate the differentiation process, thereby attacking ARMS tumor cells on multiple different biological fronts. Experiments are ongoing to test this proposed multi-faceted treatment regimen.

## MATERIALS AND METHODS

### Cell culture conditions and stable transduction of mouse primary myoblasts

Mouse primary myoblasts were isolated from 2 – 4 day old C57/Bl6 mice and cultured in proliferation media as previously described [23]. To induce differentiation, the proliferation media was removed, the cells were washed twice with PBS, and the media was replaced with 10ml of differentiation media, as previously described [23, 24]. All cells were grown on collagen-coated dishes (Becton Dickinson Labware, Bedford, MA), were passage-matched, were not used past passage nine, and were not allowed to grow past approximately 80% confluency.

Mouse primary myoblasts were stably transduced as previously described [23, 37] with the MSCV-IRES-puromycin empty vector or vector containing FLAG epitope-tagged PAX3-FOXO1 (FLAG-PAX3-FOXO1) [21, 22]. Three days post-transduction, cells were selected using puromycin, as previously described [22]. The stably transduced cells were harvested and pooled from three independent transductions to create a single population that express each construct.

### Western blot analysis

Stably transduced cells were grown to 80% confluency, harvested, and total cell extracts made, as previously described [21–23, 36]. Equal amounts of total cell lysates (12μg) were separated by 8% SDS-PAGE and analyzed by Western blot analysis using antibodies specific for Pax3, as previously described [21, 22], or MyoD (GenScript, Piscataway, NJ), Myogenein (Abcam, Cambridge, MA), Myosin heavy chain (Abcam), or GAPDH (Cell Signaling, Danvers, MA), according to manufacturers’ specifications.

### RNA extraction, cDNA library construction, and cDNA deep sequencing

Primary myoblasts stably expressing empty vector or FLAG-PAX3-FOXO1 were grown to approximately 80% confluency, differentiated for 30 minutes or 24 hours, and total RNA was isolated from the three time points (proliferating and differentiating) using the miRNeasy mini kit (Qiagen), according to the manufacturer's specifications. MicroRNA were isolated from 4μg total RNA, to generate the cDNA libraries, using the Illumina sample preparation kit according to the manufacturer's specifications. The cDNA libraries were provided a unique index identifier, allowing the clustering of several samples into a single sequencing lane, and deep sequencing analyses were performed in triplicate from three independent cell growth, RNA isolation, and cDNA library constructions.

### miRNA-seq data analysis

Raw fastq sequences were obtained from the Illumina Genome Analyzer II using the “Demultiplex” algorithm in the CASAVA 1.8.2 software (Illumina) that allows the identification of individual samples by “index sequences” contained within the adapters and introduced during the adapter ligation and amplification of the samples. miRNAKey, was used for data analysis at default settings. The pipeline clips the Illumina 3′ adaptor sequences, maps the clipped reads to miRBase and uses the Seq-EM algorithm to estimate the distribution of multiply mapped reads across the observed miRNAs. Sequences less than 16 bases after adaptor clipping were removed. The read counts obtained were then used for differential expression analysis between control and experimental samples using EBSeq from the R package with a False Discovery Rate (FDR) of 5%. We used the default ‘Median Normalization’ in EBSeq to make the counts comparable across samples. Resulting differentially expressed miRNA were analyzed using miRTarBase to identify experimentally validated target genes, with validation by at least two independent experimental methods. Finally, the differentially expressed miRNA and their target genes were analyzed using the Ingenuity Pathway Analysis (Qiagen, Valencia, CA) to identify effected pathways and biological interactions between miRNA target genes.
